# Prevalence and intensity of urinary schistosomiasis among school children in the district of Niakhar, region of Fatick, Senegal

**DOI:** 10.1186/1756-3305-7-5

**Published:** 2014-01-03

**Authors:** Bruno Senghor, Aldiouma Diallo, Seydou N Sylla, Souleymane Doucouré, Mamadou O Ndiath, Lobna Gaayeb, Félicité F Djuikwo-Teukeng, Cheikh T Bâ, Cheikh Sokhna

**Affiliations:** 1 Institut de Recherche pour le Développement, UMR 198 (URMITE), Campus International de Hann, IRD, BP 1386, CP 18524 Dakar, Senegal; 2 Département de Biologie Animale, Université Cheikh Anta Diop de Dakar, BP 5005 Dakar, Senegal; 3 Université Gaston Berger de Saint Louis, UFR Sciences Appliquées et Technologies, BP 234 Saint Louis, Senegal; 4 EPLS Biomedical Research Center, Saint-Louis, Senegal; 5 INSERM UMR 1094, Faculties of Medicine and Pharmacy, Limoges, France

**Keywords:** Epidemiology, Helminths, Infection, Intensity, Niakhar, Prevalence, Senegal, Urinary schistosomiasis

## Abstract

**Background:**

Urinary schistosomiasis is a parasitic disease that exists in all regions of Senegal. It is a major public health issue in this country. This study was carried out to determine the prevalence and intensity of this parasitosis in 12 villages of Niakhar (Fatick, Senegal).

**Methods:**

A total of 210 schoolchildren, aged 7 to 15 years, were enrolled in this study, and urine samples were examined for *Schistosoma haematobium* eggs using a standard urine filtration technique.

**Results:**

Of these children, 121 (57.6%) were found to be infected with a mean geometric count of 185 eggs per 10 ml of urine. The disease was present in all surveyed villages, and the prevalence ranged from 14.3% to 92.8%. The prevalence of infection was significantly correlated with increasing age and was higher in boys. Infection intensity was significantly higher in boys but did not significantly differ with age. Significant relationships between i) water contact or access to running water and ii) the prevalence or intensity of urinary schistosomiasis were also noted.

**Conclusions:**

The district of Niakhar is endemic for urinary schistosomiasis, with a high intensity of infection. A control program to decrease the prevalence and intensity should be implemented in this area to improve community health.

## Background

Schistosomiasis is a chronic and debilitating disease caused by digenetic trematode flatworms (flukes) of the genus *Schistosoma.* This water-dependent disease is endemic in rural areas where there is a lack of drinking water [1]. Schistosomiasis is one of the most common parasitic infections in the world, ranking second after malaria in terms of socio-economic and public health importance, especially in rural areas of developing countries [2,3]. Of the 239 million people with active *Schistosoma* infections in 2009 [4], 85% lived in sub-Saharan Africa, where approximately 112 million and 54 million were infected with urinary and intestinal schistosomiasis, respectively, and the number of persons at risk of infection is greater than 600 million [5]. Therapeutic vaccines represent an alternative to chemotherapy (praziquantel) to control this disease [6]. However, the lack of epidemiological data on parasite prevalence may hamper control interventions and the development of vaccination strategies.

In Senegal, urinary schistosomiasis has been widespread and poses a public health problem, particularly in children [7,8]. It is present in all regions of the country [9], with a mean estimated prevalence of 25% in 2003 [10]. In the Niakhar district, one previous study on the protective effect of schistosomiasis against malaria was carried out in two villages (Tukar and Diokhine), and the overall prevalence of urinary schistosomiasis was 67% [10]. As no mass treatment against this helminthiasis has been carried out in this area, the aim of the present study was to determine the prevalence and intensity of urinary schistosomiasis among school children.

## Methods

### Study area

**Figure 1:**
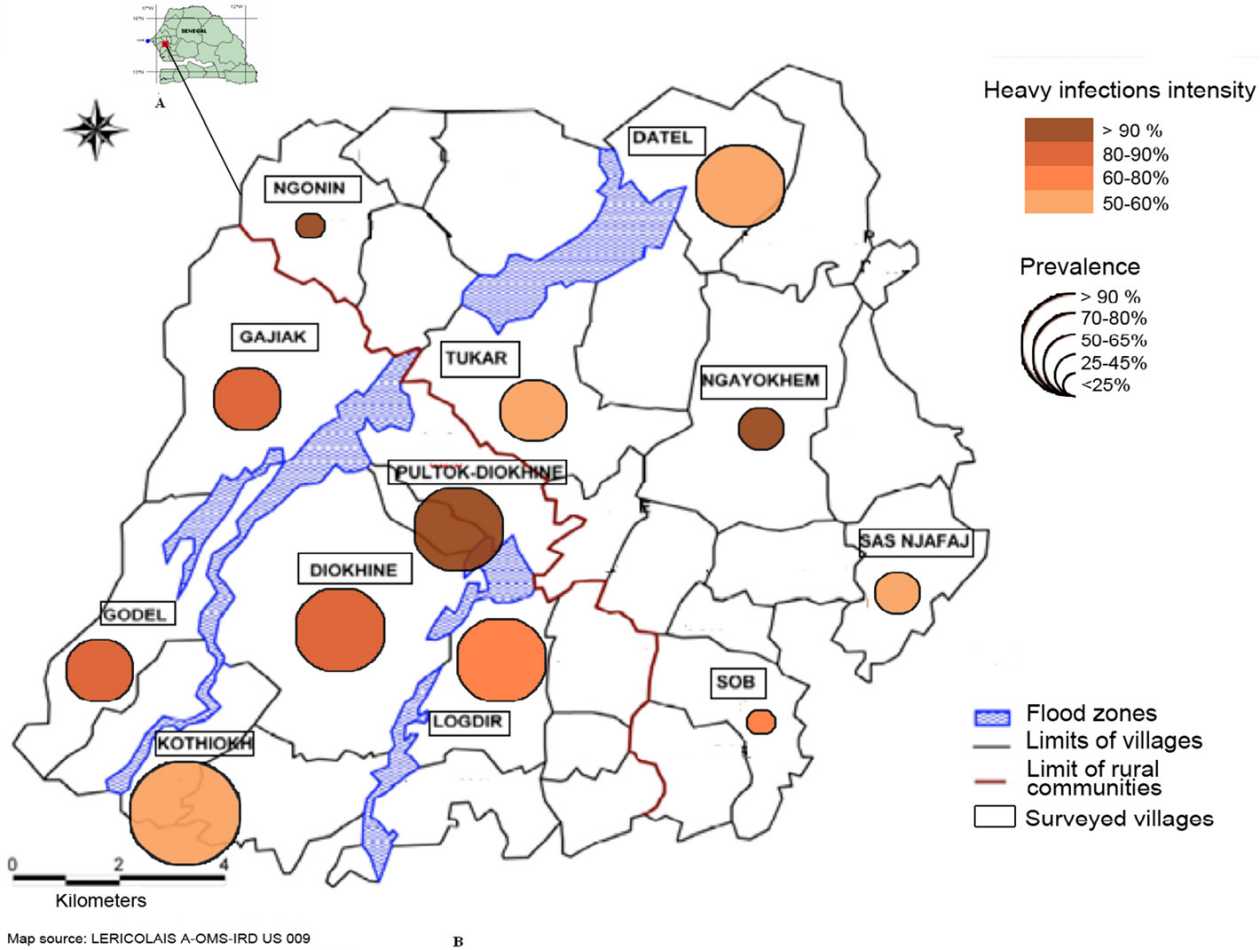
Localization of the studied area in Senegal (A) and distribution of prevalence and intensity of infection (B).

The study was carried out at Niakhar (14°30′ N, 16°30′ W), a Demographic Site Survey (DSS) located in the region of Fatick (Sine-Saloum), 135 km east of Dakar, the capital of Senegal, West Africa. The study zone (Figure 1a) was approximately 15 km long and 15 km wide and covered 230 km^2^. The climate is continental Sudan-Sahelian, with temperatures ranging from 24°C in December-January to 30°C in May-June [11]. The rainy season spans four months (July-October), and the mean annual precipitation was 506 mm from 1962 to 2010 [12]. The district includes 30 villages with a total of 43,000 inhabitants [13], mainly of the serer ethnic group (96.4%) [14]. An environmental survey of this area has mapped all of the water sources (backwater, ponds, wells and supply taps), and it showed that the two-thirds of the population had no access to tap water [11]. A more detailed description of the Niakhar district has been given elsewhere [15].

### Selection of children

All the studies in the Niakhar district were conducted with the approval of the National Ethics Committee of Senegal. Oral consent was obtained after meeting with parents of schoolchildren and chiefs of the villages. A cross-sectional descriptive and analytical study was carried out from February to June 2009, and it involved children aged 7 to 15 years. The methodology used was cluster sampling, as recommended by the World Health Organization, with samples of at least 210 subjects each, which were divided into 30 clusters of at least 7 subjects. Because we had 20 primary schools, we decided to work on 15 clusters of 14 subjects each, giving us the same sample size of 210. The sampling interval (SI) was determined by the formula SI = M/n, with M as the total number of schoolchildren in 2009 and n as the number of clusters (SI = 6.873/15 = 458). To determine the first cluster for investigation, a random draw of numbers was made between 1 and the number corresponding to the sampling interval (458). The school with the closest number of schoolchildren to the SI number was chosen first. Other schools were selected as follows: E, E + SI, E + 2 SI, E + 3 SI … (E, number of children in the first selected school). The 15 clusters were distributed over 14 villages (out of the 20 villages that had at least a primary school). A total of 210 schoolchildren were enrolled in the study.

### Sample collection and analysis

Urine samples were collected between 10:00 a.m. and 2:00 p.m., and their macroscopic aspect in transparent urine jars was examined. All samples that did not have a more or less dark yellow and a translucent aspect, without any particulates, were considered abnormal. We selected three macroscopic aspects: clear urine (urine of normal appearance and translucent), cloudy urine (urine of abnormal appearance, not translucent, with clots or suspension elements) and hematic urine (abnormal urine, non-translucent and red). Data were collected using a questionnaire whose reliability has been demonstrated in several countries for the detection of high-risk areas for schistosomiasis [16,17]. This questionnaire was given to each urine-providing child to collect information on the child’s sex, age, water contact, symptoms of urinary schistosomiasis, knowledge about the disease, and previous praziquantel treatment. A plastic urine jar of 50 ml was then given to each child with a number corresponding to the questionnaire. Urine samples were transported to the Niakhar laboratory for analysis.

Microscopic examination of each urine sample for detection of *S. haematobium* eggs was performed using the method by Plouvier *et al*. [18]. In brief, 10 ml from each sample of urine was passed through a Millipore filter (12 μm polycarbonate filter), and this filter was analyzed with a 10 × objective. The number of eggs per filter was counted, and the infection intensity was classified as light (<50 eggs/10 ml of urine) or heavy (≥50 eggs/10 ml of urine), as defined by the World Health Organization [19].

### Statistical analyses

Data were recorded using the Epi-Info, version 3.5.1 (August 13, 2008) and analyzed using STATA 11.1. The relationships between characteristics of human infection (prevalence and intensity) and other variables, such as the location of villages, sex and age of children, water contact, or access to tap water, were tested. Differences in percentages were analyzed using the χ^2^ test. The Geometric Means of Williams (GMW) was used for egg counts. The Mann–Whitney test was used for comparisons between the characteristics of human infection and the children’s sex, water contact or access to tap water, while the Kruskal-Wallis test was utilized for comparison between infection characteristics and the ages of children. Confidence intervals (CI) at 95% for GMW were estimated according to Kirkwood and Sterne [20]. A value of p < 0.05 was considered significant.

### Ethical approval

All the studies in the Niakhar district were conducted with the approval of the National Ethics Committee of Senegal, followed by the local health authority. Before the study began, the chiefs of the villages and parents were fully briefed on the objective of the study. The study was explained to each participant for their understanding and cooperation. Parents and participants were asked to consent verbally to participate in the study; only those who provided consent were enrolled and requested to prove urine samples. At the end of the study, all the children registered were treated with praziquantel according to their weight (40 mg/kg).

## Results

### Sample characteristics

The sex ratio (M/F) was 0.9, with 101 boys and 109 girls. The average age was 10.4 years (SD: 2.1 years). The individuals were divided into three age groups (7–9, 10–12 and 13–15 years), representing 37.2%, 41.4% and 21.4% of the children, respectively. Hematuria (52.4%) and dysuria (54.3%) were the most commonly reported symptoms by both infected and uninfected children. Abdominal pain (34.7%) was the least reported symptom. Macroscopic urine examination showed that clear urine (49%) was more frequent than cloudy urine (40%) or hematic (11%) urine. A few children (4.8%) had knowledge of schistosomiasis, and 5.5% had received previous treatments for this disease. A question on travel outside the Niakhar area showed that only 12% of children traveled to other regions during vacations. The others, especially the youngest, remained in their villages (Table 1).

**Table 1:** Reported symptoms and main characteristics of collected urine samples

**Symptoms and other characteristics**	**Absolute**	**Relative**
	**frequencies**	**frequencies (%)**
Haematuria reported	110	52.4
Previous treatment	6	5.5
Urinary pain	56	52.3
Abdominal pain	37	34.7
Clear urine	103	49
Cloudy urine	84	40
Macroscopic hematuria	23	11
Frequentation of water points	160	76.2
Knowledge of schistosomiasis	10	4.8
Travel outside villages	27	12.8

### Prevalence and intensity of *S. haematobium* infection

Of the 210 urine samples examined for *S. haematobium*, 121 (57.6%) were infected, with a mean geometric count (EMGC) of 185 [95% CI, 130–264] eggs/10 ml of urine, and 72.8% of the infected urine samples exceeded 49 eggs/10 ml of urine. The highest prevalence (> 90%) was found in the village of Kothiokh, and the lowest prevalence (< 25%) was found in the village of Ngonin. A significant relationship (p < 0.001) in the prevalence of infection between villages was noted. The greatest heavy infection intensities (> 90%) occurred in the villages of Ngayokhem, Ngonin and Poultok-Diokhine (Figure 1b).

### Prevalence and intensity of *S. haematobium* infection in relation to water contact and access to tap water

The prevalence of infection was greater in persons who used backwater (69.6%) than those who used ponds (53.3%). A highly significant relationship (p < 0.01) was found between the prevalence of infection and the type of water contact. The highest EGMC, 202 (95% CI, 134–314) eggs/10 ml of urine, was found in persons who use ponds. People who did not have access to tap water had the highest prevalence and EGMC. Significant differences were also noted between prevalence and access to tap water (p < 0.005), and between intensity of infection and access to tap water (p < 0.005). If only the villages with tap water are considered, the prevalence and intensity of infection were significantly higher in boys than in girls (p < 0.01). In contrast, in villages without tap water, the prevalence and intensity of infection were higher in girls, but these differences were not significant (Table 2).

**Table 2:** **Prevalence and intensity of *Schistosoma haematobium *** **witch respect to water contact and access to running water**

	**No. examined**	**No. infected**	**Egg count**
		**(%)**	**geometric**
			**mean (95% CI)**
Water contact			
Backwater	56	39(69.6)	156(77–314)
Ponds	154	82(53.2)	202(134–314)
P value			0.141
Access to running water			
Access to tap water	140	71(50.7)	176(112–277)
No access to tap water	70	50(71.4)	200(112–358)
P value			0.007
Access to tap water			
Boys	69	43(62.5)	346(209–573)
Girls	71	28(39.4)	62(30–128)
P value			<0001
No access to tap water			
Boys	32	24(75)	145(61–344)
Girls	38	26(68.4)	269(117–616)
P value			0.738

### Prevalence and intensity of *S. haematobium* infection by gender and age

Boys had a higher infection rate (66%) and EMGC (253 [162–396] eggs/10 ml of urine) than girls (49.5% and 126 [95% CI, 130–264] eggs/10 ml, respectively). Significant differences in prevalence values and intensities of infection according to sex (p < 0.05) were noted (Table 3). Boys were heavily infected regardless of age, but a significant difference (p < 0.05) was only noted between boys and girls in the 7–9 year group.

**Table 3:** **Prevalence and egg count geometric mean of *Schistosoma haematobium *** **by gender and age of schoolchildren in the study villages**

	**No. examined**	**No. infected (%)**	**Egg count**
			**geometric**
			**mean (95% CI)**
Gender			
Boys	101	67(66.3)	253(162–396)
Girls	109	54(49.5)	126(71–221)
P value			0.003
Age			
7 - 9 years	78	35(44.8)	202(102–400)
10 - 12 years	87	56(64.4)	161(96–270)
13 - 15 years	45	30(66.6)	217(100–469)
P value			0.058

The prevalence of infection increased with age, and statistical analysis indicated a significant relationship (p < 0.05) between these two variables. The EMGC decreased in the 10–12 year group, before increasing again in the 13–15 year group. No significant difference between the age of children and the infection intensity was noted (Table 3). However, if boys and girls are grouped, the intensity of infection decreased with age in girls and increased in boys. The difference between boys and girls was also significant in the 7–9 year group (p < 0.05). The variables age, gender and access to water were confronted to villages with medium and high intensity but statistical analysis showed no significant difference (p > 0.05).

## Discussion

The results of the present study show that the Niakhar district is endemic for urinary schistosomiasis. The prevalence (57.6%) and intensity of *S. haematobium* infection (185 eggs/10 ml) indicate a high risk at the community level in accordance with the WHO definition [19]. This high rate of egg excretion might be due to the age group studied because children from 7 to 14 years old are the most important egg shedders [21,22]. In addition, only 5.5% of children had received treatment for schistosomiasis, which would explain the rates observed. This finding is also attributable to intense water contact activities in the area. The same observation was also made in Senegal (in the region of Bignona, Casamance [23], at Barkedji in the department of Linguere [24]) and in several Nigerian villages around the Gusau dam, Zamfara [25]. Communities in the study area of Niakhar are rural, and most of the villages depend on backwater and ponds for their water needs, such as bathing, swimming, fishing and other domestic uses. These water bodies provide natural water sources and also serve as habitats to intermediate hosts (bulinids) and schistosome parasites. These water bodies constitute the main transmission foci of *S. haematobium* in the communities and are distributed throughout the area. These conditions make it certain that the people will continue to be infected and re-infected because no intervention strategy has been implemented in the area.

However, the differences found in the prevalence and intensity of infection between the villages investigated could be attributed to the fact that people living in Datel, Gajiak, Godel and Kothiokh were dependent on backwater as their principal water source during the rainy season. The high prevalence in these villages reflects the higher level of exposure and dependence of these inhabitants on backwater, which persists during the dry season [11,14]. In addition, these villages did not have access to tap water [11]. The low prevalence observed in the other villages might be due to the fact that they depended on ponds, which dried more rapidly than backwater, and to the presence of running water for their daily uses. This observation agrees with other reports conducted in Nigeria [25,26] where variability and epidemiology of the disease were attributable to water-contact patterns. It is also similar to the observations by Nkengazong *et al*. [27], who showed in south west Cameroon that villages without pipe-borne water access maintained a high level of infection. However, in some villages with running water in the Niakhar district, the prevalence was also high. This situation might be explained by other factors such as the proximity with ponds, lack of health education and poor hygiene.

The higher prevalence in boys than in girls confirms other reports for human infection in Senegal [24,28] and in several different localities in West African countries [29,30]. However, this result does not agree with the reports by Dabo *et al*. [31] in Mali and Ahmed *et al*. [32] in central Sudan, who found similar prevalence in boys and girls. EGMC was strongly correlated with gender, with boys being more heavily infected than girls. This difference noted in the Niakhar district may be due to cultural, behavioral and social factors. Indeed, during the rainy season, boys participate in various activities, such as swimming, washing domestic animals, fishing, etc., that create frequent and prolonged contact with water sources. In contrast, girls are restricted socially from water contact activities such as swimming and bathing. They also go to backwater and ponds for the washing of clothes or fetching water for domestic work. In villages with running water, girls usually stay at home and generally use tap water for housework, thus reducing their contact with other water sources. Boys thus constitute a high risk group for urinary schistosomiasis in Niakhar.

The highest prevalence values of urinary schistosomiasis were recorded in the 10–12 and 13–15 year groups. These age classes are most likely responsible for schistosomiasis transmission in the area. The increase of prevalence with increasing age of children was also noted in other African countries. In Burkina Faso, Poda *et al*. [33] found a significant difference between three age groups (7–9, 10–12, and 13–16 years). A similar result was also reported by Briand *et al*. [10] in two villages from the same district of Niakhar. In the present study, the percentage of infected schoolchildren in the 10–12 year group was practically equal to that noted in the 13–15 year group, and this last finding agrees with several reports [22,34] where there was a peak at 10–14 years. No significant difference was observed between the age of children and the intensity of infection. This finding also agrees with previous reports [34,35] where the intensity of infection did not show any significant difference with the age of children.

If children are grouped by age and sex, the intensity of *S. haematobium* infection increased with age in boys but decreased in girls. According to Gryseels *et al*. [1], the decline of intensity of infection among older children in some populations is due to a decreased contact with infected water [1]. In the Niakhar district, the decrease in infection observed among older girls might be explained by their seasonal migration to urban areas during the rainy season to seek employment as domestic workers [14]. Boys stay in the villages during the rainy season, thus maintaining a high intensity of egg parasites, most likely due to seasonal re-infection. A snail survey is needed to assess the role of water sources in the transmission of *S. haematobium*. The implementation of a control program in this area to decrease prevalence and intensity would also be highly suitable.

## Conclusions

The results obtained show that the Niakhar area is endemic for urinary schistosomiasis. Health education and large-scale chemotherapy for all schoolchildren to decrease the prevalence and intensity of infection would be highly suitable. All the villages of the Niakhar district need access to piped water to reduce contact with infected waters.

## Competing interests

The authors declare that they have no competing interests.

## Authors’ contributions

Conceived and designed the study: AD, CTB and BS. Performed field activity: AD and BS. Performed the experiment: BS. Analyzed the data: SNS, FFD-T, LG and BS. Wrote the paper: BS, SD, FFD-T., LG and MON. Supervised the study: AD, CTB and CS. All authors approved the final version of the manuscript.
